# Feeding ecology of an Amazonian electric knifefish under altered flood‐pulse dynamics caused by hydroelectric damming

**DOI:** 10.1111/jfb.70335

**Published:** 2026-01-27

**Authors:** Ana F. V. N. M. Costa, Antonio A. Jardim, Erival G. Prata, Rafael R. Gusmão, Angelo J. Faro Júnior, Alan S. Fonseca, Danielly T. Hashiguti, Luciano F. A. Montag, Tiago M. S. Freitas

**Affiliations:** ^1^ Laboratório de Ecologia e Conservação, Instituto de Ciências Biológicas Universidade Federal do Pará Belém Brazil; ^2^ Programa de Pós‐Graduação em Zoologia Universidade Federal do Pará Belém Brazil; ^3^ Programa de Pós‐Graduação em Ecologia Universidade Federal do Pará Belém Brazil; ^4^ Núcleo de Ecologia Aquática e Pesca da Amazônia Universidade Federal do Pará Belém Brazil; ^5^ Laboratório de Zoologia, Faculdade de Ciências Naturais Campus Universitário do Marajó‐Breves, Universidade Federal do Pará Breves Brazil

**Keywords:** *Archolaemus janeae*, diet, Gymnotiformes, hydroelectric, Xingu River

## Abstract

This study evaluated the effects of the controlled flood pulse on diet composition, trophic niche breadth and feeding intensity of *Archolaemus janeae*, an electric knifefish species with a restricted distribution in the Amazon Basin. Monthly samples were collected from December 2020 to November 2021 in the Volta Grande stretch of the Xingu River, and stomach contents of 134 specimens were analysed. The diet was predominantly composed of aquatic insect larvae, with a notable occurrence of terrestrial plant fragments. Hydrological variation affected diet composition, trophic niche breadth and feeding intensity, indicating temporal shifts in resource use associated with changes in river flow. Feeding intensity was higher during filling and flood periods than during the ebb phase, suggesting that the reduction and regulation of the flood pulse by the Belo Monte Hydropower Plant influenced the diet composition by *A. janeae*. This study provides the first data on the feeding ecology of this species, highlighting the role of habitat heterogeneity in sustaining fish populations and underscoring the potential impacts of hydropower development on the feeding ecology of Amazonian fishes. Such information is essential for the conservation of *A. janeae* populations in the Xingu River Basin.

## 1 INTRODUCTION

The flood pulse is a seasonal hydrological phenomenon characteristic of floodplain forest areas, such as the *várzeas* of the Amazon basin, and plays a fundamental role in the ecological dynamics of these ecosystems (Freitas et al., 2011; Junk et al., 1989). During the high‐water period, rising water levels inundate forests adjacent to the main river channel, thereby increasing connectivity between aquatic and terrestrial habitats (Goulding, 1980; Junk et al., 1989). This spatial expansion creates a diversity of habitats that provide foraging opportunities, reproductive sites and shelter for numerous fish species (Junk et al., 1989; Lowe‐McConnell, 1999). Conversely, during the dry season, when water levels recede, fish concentrate and become restricted to habitats such as river channels and rapids (Barbosa et al., 2018; Goulding, 1980).

This hydrological dynamic strongly influences the feeding ecology of fishes: during high‐water periods, the input of allochthonous resources (e.g. plant materials, terrestrial invertebrates and detritus) increases, integrating terrestrial production into aquatic food webs (Correa & Winemiller, 2014; Dary et al., 2017; Freitas et al., 2022). In contrast, during low‐water periods, the reduction of terrestrial‐derived inputs leads to a diet predominantly based on autochthonous resources, such as organic matter, algae and aquatic macroinvertebrates (Cunico et al., 2002).

Given this strong spatial–temporal variation in resource availability, assessing fish trophic ecology through stomach content analysis represents a valuable approach to understanding feeding interactions between species and their environment (Amundsen & Sánchez‐Hernández, 2019). This method allows direct identification of ingested items, enabling the trophic classification, qualitative and quantitative estimation of diet composition, analysis of dietary niche breadth and detection of seasonal shift in resource use (Bianchi‐Costa et al., 2023). In systems influenced by the flood pulse, stomach content analysis reveals how fish diets respond to fluctuations in water levels, reflecting changes in the availability of autochthonous and allochthonous resources across hydrological periods (Penha et al., 2024, 2025; Prata et al., 2025). It also provides critical insights into the ecological role of each species within the food web (Correa & Winemiller, 2018; Pereyra et al., 2021).

However, this ecological balance can be disrupted by anthropogenic interventions associated with hydropower development, which alter the natural flow regimes, reduce habitat connectivity and modify resource availability, thereby directly impacting the trophic ecology of local fish assemblages (Barros et al., 2020; Seabra et al., 2025; Timpe & Kaplan, 2017). A notable case is the Belo Monte Hydroelectric Power Plant (UHE Belo Monte) in the Volta Grande do Xingu (VGX), Xingu River basin (Brazil). Despite modifications to its original design aimed at mitigating social and environmental impacts by adopting a run‐of‐river operation (Baird et al., 2025), the plant has profoundly altered the hydrological regime, reducing downstream discharge, negatively affecting the flood pulse, fishery yields and the ecological integrity of aquatic biota (Castro‐Diaz et al., 2018; Stickler et al., 2013). In this context, studies of fish feeding ecology are essential because they allow assessment of the ecological consequences of river damming, thereby supporting conservation and management strategies for impacted species (Penha et al., 2024, 2025; Prata et al., 2025).

Among the ichthyofauna of the Xingu River, the electric knifefish *Archolaemus janeae* (Vari et al., 2012) stands out. This species, with a restricted distribution in the Amazon basin (upper Tapajós River and middle Xingu River), belongs to the family Sternopygidae (order Gymnotiformes) (Rodrigues et al., 2021; Vari et al., 2012). It exhibits an elongated, hydrodynamic body adapted for swimming in freshwater environments, particularly over benthic substrates (Vari et al., 2012), with an average total length of 38 cm in Xingu River populations (Giarrizzo et al., 2015). Despite its restricted range and distinctive morphology, the ecology and feeding habits of *A. janeae* remain poorly studied. Preliminary observations suggest a carnivorous diet, feeding on aquatic insect larvae, particularly those of Chironomidae (Vari et al., 2012). This pattern is consistent with other Sternopygidae species, which exhibit benthivorous and insectivorous diets, highlighting their role in structuring food webs and contributing to the ecological dynamics of Amazonian aquatic systems (Campos et al., 2021). As an intermediate consumer in benthic food webs, *A. janeae* may play an important role in regulating macroinvertebrate populations and transferring energy across trophic levels (Giora et al., 2014).

Beyond their morphological features, these gymnotiform fishes possess specialized electrogenic adaptations used for communication and prey detection in low‐visibility environments (Nanjappa et al., 2000). The selection of *A. janeae* as a model species in this study is justified by the scarcity of ecological information available on its feeding ecology, particularly in areas subject to severe environmental alterations such as those caused by river damming. Thus, the evaluation of trophic metrics provides an opportunity to infer how changes in the flood pulse affect trophic plasticity in a controlled‐flow system (Abujanra et al., 2009; Bianchi‐Costa et al., 2023).

Accordingly, this study aimed to assess the effects of the flood pulse on the trophic ecology of *Archolaemus janeae* in the Volta Grande region of the middle Xingu River, within a reduced‐flow stretch directly impacted by the Belo Monte Hydropower Plant. Specifically, we sought to evaluate how flow regulation influences diet composition, trophic niche breadth and feeding intensity in this species. We hypothesize that variations in hydrological regimes alter the availability and diversity of food resources, leading to a broader trophic niche and greater feeding intensity during high‐water periods due to increased inputs of allochthonous items (Bianchi‐Costa et al., 2023; Silva et al., 2021). Conversely, during low‐water periods, when resource availability is more limited, we expected lower diet diversity, reduced niche breadth and lower feeding intensity, reflecting a predominance of autochthonous items (Petry et al., 2003).

## 2 MATERIALS AND METHODS

### 2.1 Study area

**FIGURE 1:**
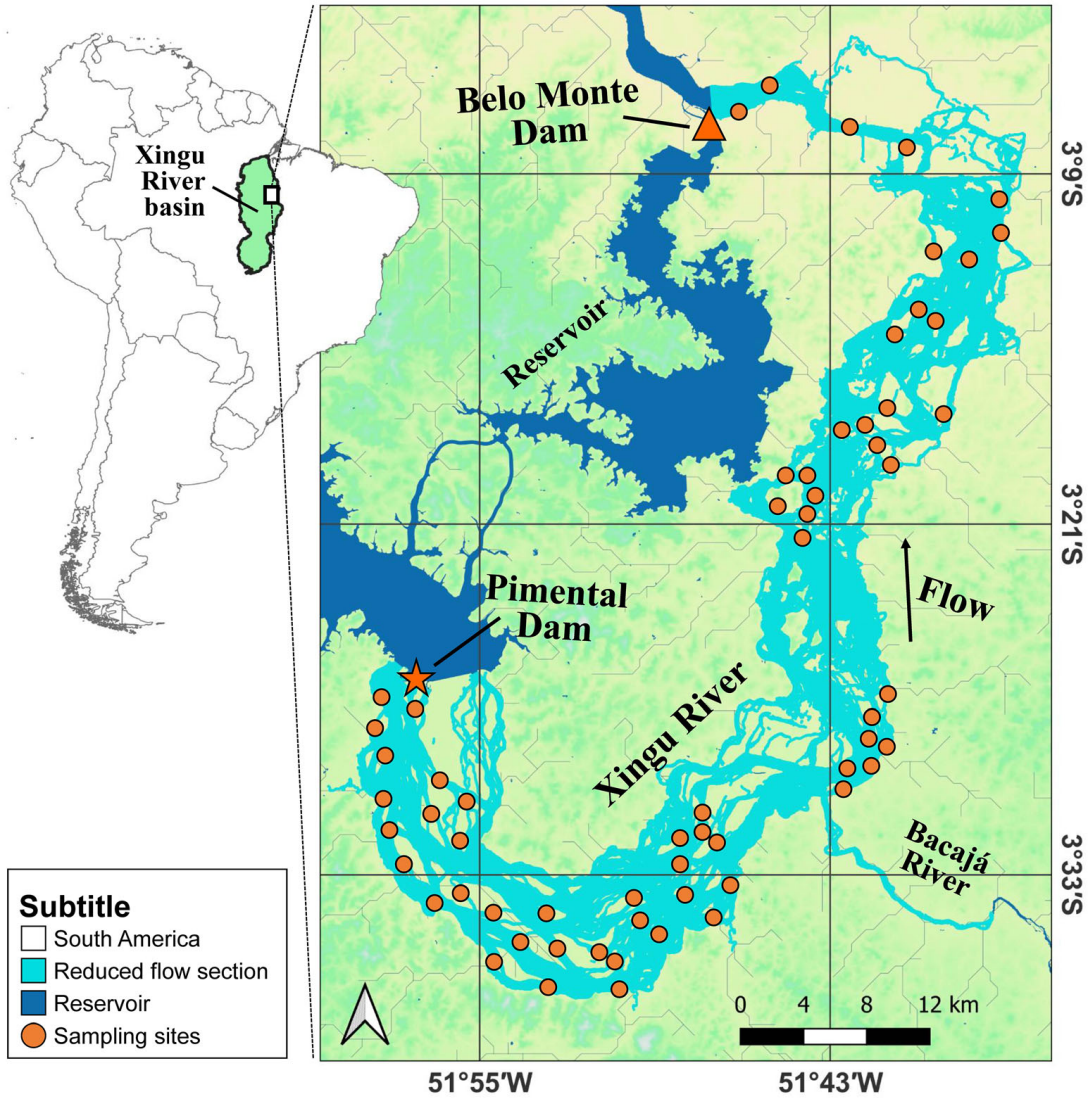
Sampling sites of *Archolaemus janeae* in the reduced‐flow stretch of the Volta Grande do Xingu, middle Xingu River (Pará, Brazil).

The study was conducted in the reduced flow stretch (RFS) of the Belo Monte Hydropower Plant, located in the Volta Grande do Xingu, middle Xingu River (Figure 1). This section corresponds to the stretch between the Pimental and the Belo Monte dams and has been characterized by a controlled hydrological regime since the implementation of this hydropower complex in 2016 (Bertassoli Jr et al., 2021).

The region has a hot and humid tropical climate, classified as Am under the Köppen‐Geiger system (Peel et al., 2007), with a mean annual temperature ranging from 17.5 to 24.5°C and relative humidity between 84% and 86%. Annual precipitation varies from 2066 to 2379 mm, with a pronounced rainy season from December to May (Barbosa et al., 2018; Sabaj et al., 2024). The physicochemical characteristics of the Xingu River within the RFS include clear waters with transparency ranging from 1 to 5 m, rocky outcrops, predominantly sandy substrates and Amazonian forest interspersed with open areas (Barbosa et al., 2018; Fitzgerald et al., 2018).

Before the dams' construction, the Xingu River exhibited a natural flood pulse with well‐defined seasonal variations between flood and dry periods, which were fundamental for sustaining local biodiversity and ecological processes (Sabaj et al., 2024). Since the dams' operation, the RFS has been subject to artificial flow regulation, with discharge reduction of up to 80% below the historical annual average recorded for the Volta Grande do Xingu (Penha et al., 2025; Zuanon et al., 2019).

Hydrological records indicated marked seasonal fluctuations during the study, with mean daily discharges ranging from 8215.9 m^3^ s^−1^ in March (flood peak) to 707.8 m^3^ s^−1^ in October (dry season minimum). Following this seasonal regime, we distinguished four distinct hydrological periods: filling (December to February), flood (March to May), ebb (June to August) and dry seasons (September to November) (Freitas et al., 2022; Lima et al., 2023; Penha et al., 2024). In this study, hydrological periods were adopted as a proxy for the flood pulse, with the rising and flood stages corresponding to peak water levels, and the receding and dry stages reflecting the lowest discharges.

### 2.2 Sampling and processing of biological material

Sampling was conducted monthly between December 2020 and November 2021 in backwater habitats. Fish were captured using rectangular gillnets, 20 m long and 1 m high, made of monofilament nylon line with mesh sizes of 2, 3, 4, 5, 6, 7, 8, 10, 12, 16 and 18 cm between opposite knots. Three sets of nets were deployed daily and left in the water for 4 h (from 17:00 to 21:00), coinciding with the species' activity both during the day and at night (Vari et al., 2012).

Captured specimens were anaesthetised with benzocaine (0.1 g L^−1^) and subsequently euthanized following the guidelines of the National Council for the Control of Animal Experimentation. Standard length (SL, cm) and total mass (TM, g) were recorded for each specimen. Stomachs were removed through a longitudinal incision from the urogenital opening to the base of the head, preserved in 70% ethanol and transported to the Laboratory of Ecology and Conservation at the Federal University of Pará (UFPA).

Voucher specimens were fixed in 10% formalin for 48 h, transferred to 70% ethanol and incorporated into the ichthyological collection of the Zoological Museum at UFPA. All procedures for capture, collection and transport of biological material were authorized by the Brazilian Institute of Environment and Renewable Natural Resources (SISBIO permit No. 1267/2020) and approved by the Animal Use Ethics Committee of UFPA (CEUA No. 8.293.020.418).

In the laboratory, stomach contents of *A. janeae* were analysed under a stereomicroscope with up to 32× magnification. Food items were identified to the lowest possible taxonomic level using specialized literature (Buckup, 2007; Hamada et al., 2014; Mugnai, 2010) and through consultation with experts. Each food item was weighed individually on a precision electronic balance (0.0001 g). Items were subsequently grouped into five trophic categories: aquatic insects, fishes, inorganic detritus (autochthonous), terrestrial insects and terrestrial plants (allochthonous).

### 2.3 Data analysis

A total of 177 *A. janeae* specimens were captured, with standard lengths ranging from 13.5 to 40 cm (mean ± standard deviation = 25.1 ± 4.5 cm) and weights between 8.7 and 68 g (20.5 ± 9.1 g). Of these, 57 individuals had empty stomachs, while the remaining 120 were used for analyses of diet composition, niche breadth and feeding intensity. Diet composition was assessed using frequency of occurrence (FOi%) and percentage of mass (Mi%), where FOi% represents the proportion of stomachs containing a given food item relative to the total number of stomachs analysed (Hyslop, 1980), while Mi% represents the mass contribution (g) of each food item relative to the total stomach contents mass (Hynes, 1950). Both parameters were combined to calculate the alimentary index (Ai%) following the modified formula of Kawakami and Vazzoler (1980): Ai% = [(FOi% × Mi%)/(∑FOi% × Mi%)] × 100, where Ai% indicates the relative importance of each food item by simultaneously integrating occurrence and mass, providing a more robust representation of the main resources used by the species. The Ai% was calculated for the entire sample and separately for each hydrological period. To test for differences in diet composition among hydrological periods, we applied permutational multivariate analysis of variance (PERMANOVA) using the *adonis2* function from the *vegan* package (Oksanen et al., 2022) in RStudio (R Core Team, 2025). In this analysis, Ai% values grouped by collection day within each hydrological period were considered as sampling units.

We applied a non‐metric multidimensional scaling (NMDS) ordination based on the Bray–Curtis dissimilarity index. The NMDS was conducted with two dimensions (*k* = 2) and the ordination quality was assessed using the stress value, which measures the degree of correspondence between the original dissimilarities and their representation in the reduced dimensional space. The resulting ordination was visualized in a scatterplot with 95% confidence ellipses for each hydrological period.

Trophic niche breadth across hydrological periods was evaluated using permutational analysis of multivariate dispersion (PERMDISP) with the *vegdist* and *betadisper* functions, also from the *vegan* package. This method estimates the average distance of individuals to the group centroid (i.e. hydrological period), reflecting within‐group dietary variation and thus the population's trophic niche breadth (Correa & Winemiller, 2014). For both PERMANOVA and PERMDISP, pairwise tests were conducted when significant differences were detected in the global models (Zar, 2010).

Feeding intensity was assessed using the repletion index (RI), calculated for each individual following Zavala‐Camin (1996): RI = (MI/Mt) × 100, where MI is the stomach contents mass and Mt is the total body mass. These values were tested for normality and homoscedasticity; since assumptions were not met, a nonparametric statistical approach was adopted. Variation in RI among hydrological periods was tested using the Kruskal–Wallis test (*kruskal. test* function, *stats* package, RStudio). When significant differences were found, post hoc pairwise multiple comparisons were performed using Dunn's test to identify which periods differed in feeding intensity. For all statistical analyses, the significance level was set at 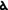 = 0.05 (Zar, 2010).

## 3 RESULTS

**FIGURE 2:**
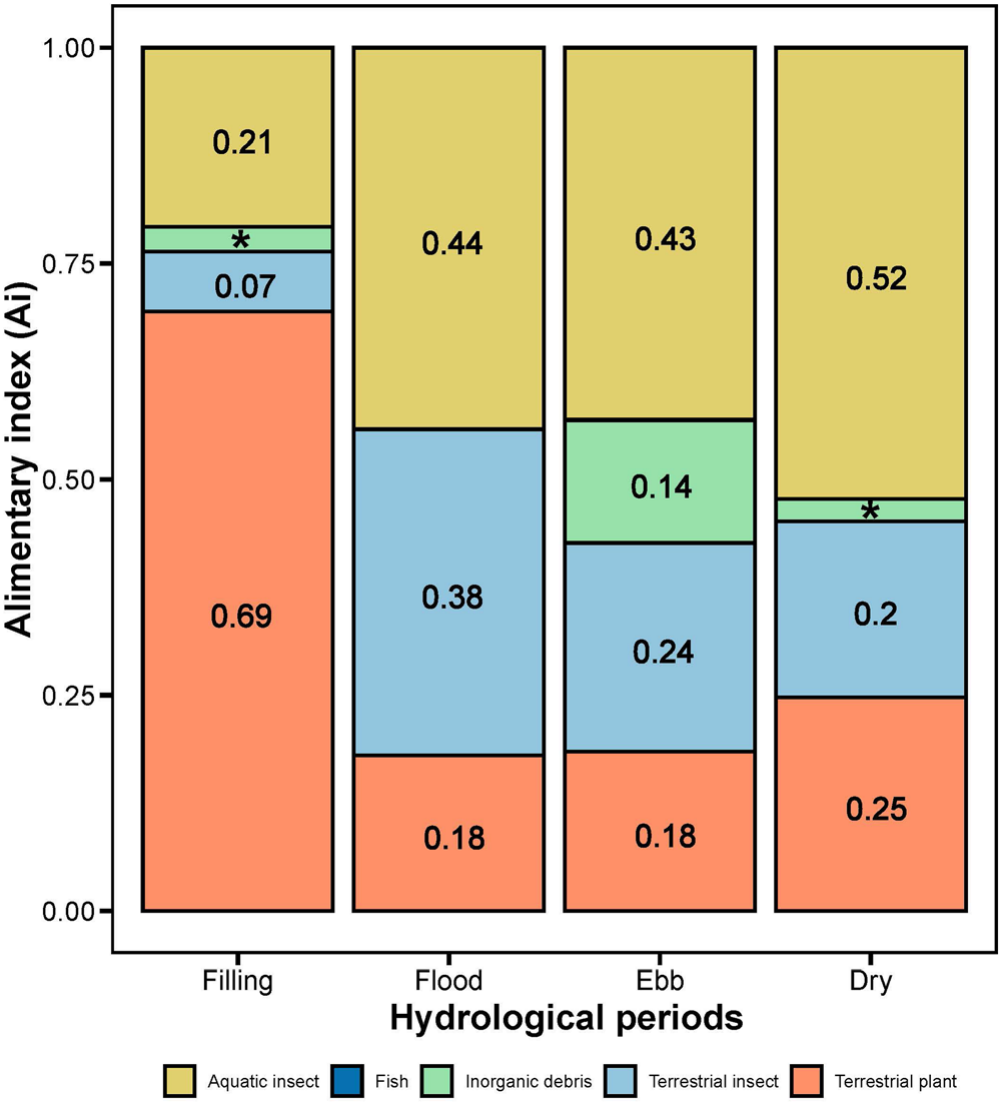
Alimentary index (Ai%) of the diet of *Archolaemus janeae* across different hydrological periods in the middle Xingu River, eastern Amazonia, Brazil. *Represents values <3% of contribution.

The diet of the *A. janeae* population comprised 14 food items, with the most important being fragments of terrestrial plants (33% Ai), followed by fragments of terrestrial insects (22% Ai) and Odonata larvae (Gomphidae) (14% Ai). Detailed information on the food items and their relative importance in each hydrological period is presented in Table 1. The most representative trophic categories were aquatic insects (40% Ai), followed by terrestrial plants (33% Ai) and terrestrial insects (22% Ai). The remaining categories together accounted for only 5% (Ai%; Figure 2).

**TABLE 1:** Alimentary index (Ai%) of food items and trophic categories in the diet of *Archolaemus janeae* collected monthly and across different hydrological periods in the middle Xingu River, eastern Amazonia, Brazil.

Foods items	2020						2021						IAi%
	Filling			Flood			Ebb			Dry			
	Dec, *n* = 2	Jan, *n* = 2	Feb, *n* = 3	Mar, *n* = 2	Apr, *n* = 3	May, *n* = 3	Jun, *n* = 13	Jul, *n* = 15	Aug, *n* = 29	Sep, *n* = 29	Oct, *n* = 5	Nov, *n* = 14	
Autochthonous													
Aquatic insect													0.40
Ephemeroptera			0.01		0.15		<0.01	0.03	0.01	0.03	<0.01	<0.01	
Leptophlebiidae	<0.01	0.30	0.01	<0.01			<0.01			<0.01		<0.01	
Odonata larvae			0.28			0.01	0.19		0.02	<0.01	0.02	0.13	
Libellulidae								<0.01	<0.01			0.08	
Gomphidae	<0.01	<0.01		0.25	0.66	0.09	0.29	0.13	0.16	0.01		0.06	
Trichoptera							<0.01	0.01	0.02	0.40		0.01	
Diptera	0.02		<0.01		0.16	<0.01	<0.01	0.17	0.25	0.25	0.51	0.06	
Diptera pulp								<0.01	<0.01	<0.01			
Belostomatidae								0.01					
Perlidae												<0.01	
Fish													<0.01
Fish scale								<0.01				<0.01	
Inorganic debris													0.05
Sediment		0.09	<0.01	<0.01	<0.01	<0.01	<0.01	0.43	<0.01	0.02	<0.01	0.06	
Allochthonous													
Terrestrial insect													0.22
Insect fragment	0.14	0.02	0.05	0.22	0.03	0.89	0.06	0.18	0.49	0.11	0.47	0.04	
Terrestrial plant													0.33
Plant fragments	0.84	0.59	0.65	0.53	<0.01	0.01	0.46	0.05	0.05	0.18		0.56	

*Note*: *n* = number of stomachs containing food items.

**FIGURE 3:**
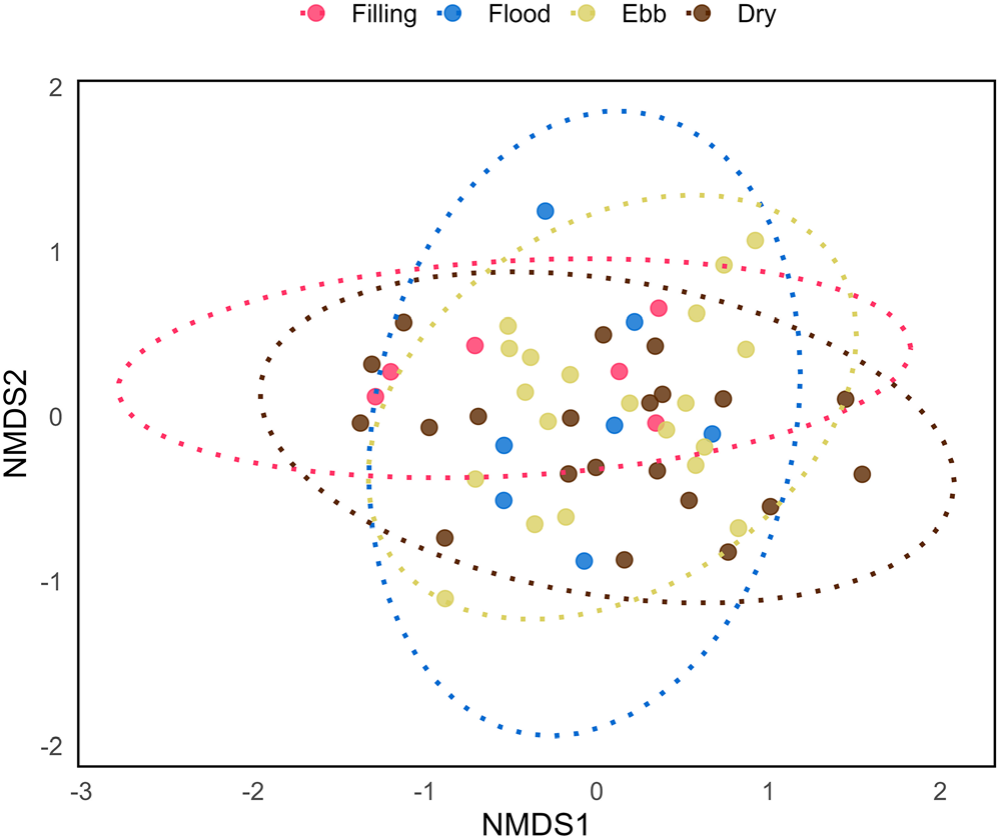
Non‐metric multidimensional scaling (NMDS) ordination showing diet composition of *Archolaemus janeae* across hydrological periods in the Xingu River. Each point represents a sample and ellipses indicate 95% confidence intervals around group centroids for each period. The two‐dimensional NMDS solution yielded a stress value of 0.12, indicating a satisfactory representation of Bray–Curtis dissimilarities.

The NMDS ordination showed good fit (stress = 0.12), with substantial overlap but clear trends of separation among hydrological periods (Figure 3). Accordingly, we observed significant differences in the diet composition of *A. janeae* across hydrological periods (PERMANOVA; pseudo‐*F* = 1.60, *df* = 3, *p* = 0.01) (Table 2). Pairwise comparisons indicated differences between the filling and ebb periods (*p* = 0.04) and between the flood and ebb periods (*p* = 0.03). The most important items for each period were terrestrial plants (79%), predominantly during the filling period, terrestrial insects (IAi = 69%) during the flood period and aquatic insects during the ebb and dry periods (IAi = 47% and IAi = 56%, respectively).

**TABLE 2:** Results of statistical analyses comparing the diet composition (PERMANOVA) and trophic niche breadth (PERMDISP) of *Archolaemus janeae* across different hydrological periods in the middle Xingu River, eastern Amazonia, Brazil.

Hydrological periods	PERMANOVA		PERMIDISP	
	Pseudo‐*F* = 1.60, *p* = 0.01		*F* = 5.12, *p* = <0.01	
	*F* value	*p* value	*t* value	*p* value
Filling vs. flood	1.2	0.27	0.02	0.87
Filling vs. ebb	1.77	0.04*	5.4	0.02*
Filling vs. dry	1.33	0.12	9.84	0.01*
Flood vs. ebb	1.87	0.03*	6.33	0.01*
Flood vs. dry	1.47	0.08	9.48	0.01*
Ebb vs. dry	1.59	0.06	0.72	0.37

*Note*: * indicates statistically significant differences (*p* < 0.05).

**FIGURE 4:**
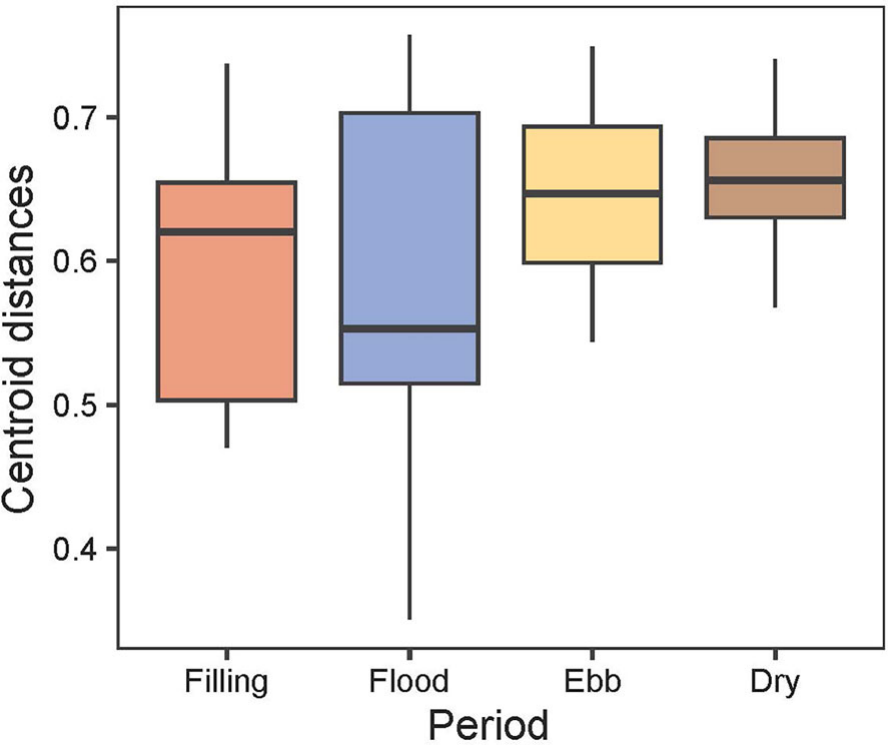
Trophic niche breadth of *Archolaemus janeae* across hydrological periods in the reduced‐flow stretch of the middle Xingu River. The horizontal line inside each box represents the median, the box boundaries indicate the first and third quartiles, and the whiskers show the minimum and maximum values.

The effect of the flood pulse on trophic niche breadth among hydrological periods was also detected (PERMDISP; *F* = 5.12, *df* = 3, *p* < 0.01) (Table 2). Differences were evident when comparing the mean centroid distances to for the flood (centroid distance; dc = 0.58) with those for the ebb (0.65, *p* = 0.02) and dry (0.66, *p* < 0.01), as well as between filling (dc = 0.59) and both ebb (*p* = 0.02) and dry (*p* < 0.01) (Figure 4). These results suggest a generalist feeding pattern for the population, with the exploitation of different resources across hydrological periods.

Feeding intensity, represented by the RI, ranged from 0.001 to 0.86 (0.11 ± 0.18). However, no significant differences were observed in the amount of food ingested by *A. janeae* specimens among hydrological periods (Kruskal–Wallis; *χ* ^2^ = 7.92, *df* = 3, *p* = 0.06).

## 4 DISCUSSION

The stomach content analysis of the *Archolaemus janeae* population revealed a diet predominantly composed of aquatic insect larvae, confirming an insectivorous feeding habit. Additionally, fragments of terrestrial plants were recorded, indicating the opportunistic use of allochthonous resources available in the environment. Diet composition and trophic niche breadth varied across hydrological periods, supporting our hypothesis and suggesting that the species adjusts its feeding spectrum in response to seasonal changes in the flood pulse. On the other hand, mean feeding intensity remained constant across periods, contrary to the expectation of greater consumption during low‐water conditions. This pattern may reflect a stable foraging strategy or structural constraints of the environment resulting from the flow regulation imposed by the Belo Monte Hydropower Plant, which may dampen the natural variation in resource availability typically observed under unregulated flood pulse conditions (Arantes et al., 2019). Overall, these findings indicate a relatively constant quantitative consumption pattern across hydrological periods, possibly as a response to hydrological alteration.

The dietary composition of *A. janeae* across hydrological periods reveals a trophic strategy characterized by high flexibility and responsiveness to spatial and temporal variation in resource availability (Ferreira et al., 2024; Winemiller, 1996), a pattern previously described for floodplain fishes (Lima et al., 2023; Mérona & Rankin‐de‐Mérona, 2004; Röpke et al., 2014). The predominance of allochthonous resources during the filling and flood periods, such as plant material and terrestrial insects, reinforces the role of flooded forests as key resource providers for marginal‐habitat species, as documented for fishes from Amazonian floodplains and other tropical flooded forests (Correa & Winemiller, 2018; Duarte et al., 2019; Rayner et al., 2009). For example, benthic species from the Purus River, including gymnotiform fish, consumed large amounts of allochthonous material during high water, reflecting the high availability of these resources and strengthening the trophic linkage between aquatic fauna and the riparian zone in floodplain environments (Correa & Winemiller, 2018; Junk, 2005).

During the ebb and dry periods, the increased consumption of aquatic insects appears to be associated with the higher concentration of benthic prey (Rayner et al., 2009). The greater intake of aquatic insects during water‐level decline represents a recurrent trophic response under different hydrological contexts in the Amazon Basin (Duarte et al., 2019; Oliveira et al., 2025; Zuanon et al., 2019). Similar patterns have also been reported in tropical systems outside the Amazon, such as the Mulgrave River in Australia, where benthic fishes exhibited high sensitivity to the availability of invertebrates across hydrological periods (Rayner et al., 2009). In that study, despite a marked reduction in the ingestion of benthic invertebrates during high water, fish displayed trophic adjustments and spatial displacements to maximize access to preferred resources, highlighting dietary plasticity as an adaptive mechanism in environments subject to pronounced seasonal fluctuations in prey availability (Pyke & Starr, 2021; Rayner et al., 2009).

The presence of terrestrial plant material, commonly recorded in the stomach contents of floodplain fishes, demonstrates the species' capacity to exploit resources derived from increased lateral river connectivity (Correa & Winemiller, 2018; Junk, 2005). The expansion of the feeding spectrum, even with items of lower energetic value, represents an adaptive response to the sudden increase in resource diversity and abundance during the flood phase (MacArthur & Pianka, 1966; Pyke & Starr, 2021). Moreover, given the spatial heterogeneity characteristic of riverine environments (Woodward & Hildrew, 2002), the incorporation of resources of different origins may reflect a flexible response to the rapid shifts in food availability promoted by hydrological dynamics (Barbosa et al., 2018; Freitas et al., 2011, 2022). This pattern likely resulted in the expansion of niche breadth during high and receding water, indicating opportunistic and diversified resource exploitation, and reflecting the species' capacity to broaden its feeding spectrum under seasonal environmental variation (Blanchette et al., 2014; Cardoso & Couceiro, 2017; Medeiros & Arthington, 2014).

The absence of a flood pulse effect on feeding intensity suggests a flexible foraging strategy that maintains relatively constant food intake across hydrological periods. This pattern may represent an adaptation typical of species inhabiting strongly seasonal environments, where sustaining a minimum continuous intake is essential to meet energetic and physiological demands regardless of fluctuation in resource (Dill, 1983; O'Gorman et al., 2021). From the perspective of optimal foraging theory (MacArthur & Pianka, 1966), stability in feeding intensity may indicate that *A. janeae* adjusts its selectivity and foraging effort according to resource abundance and accessibility, exploiting more profitable prey when available and resorting to less preferred items during periods of scarcity, particularly in hydrologically dynamic environments (Blanchette et al., 2014; Pyke & Starr, 2021; Röpke et al., 2014).

Alterations in the hydrological regime caused by large dams, such as the Belo Monte Hydropower Plant in the Volta Grande do Xingu, represent a direct threat to the ecological processes sustaining fish feeding (Penha et al., 2025; Prata et al., 2025; Seabra et al., 2025). Studies conducted in the Amazon and elsewhere have demonstrated that flow regulation directly alters fish feeding patterns (Baird et al., 2025). Common responses include reduced prey diversity and availability, diet simplification and increased dietary generalism as compensatory mechanisms for resource loss (Luo et al., 2024; Ru et al., 2022; Wang et al., 2016). These dietary shifts are directly linked to the physical modification of riverine habitats, as decreased connectivity between the main channel and floodplains, combined with reduced amplitude and duration of flooding, limits access to marginal habitats rich in aquatic invertebrates and allochthonous material, key dietary components for many fish species (Agostinho et al., 2008).

The consequences of hydrological alteration are even more critical for species with restricted distributions, such as *A. janeae* (Rodrigues et al., 2021; Vari et al., 2012). The spatial limitation imposed by its narrow geographic range, combined with the changes in flow regime resulting from the Belo Monte Dam, exposes this population to less favourable and less predictable environmental conditions (Agostinho et al., 2004; Fitzgerald et al., 2018). This scenario forces individuals to adopt more generalist diets, as observed in our results, reflecting trophic plasticity that, while ensuring short‐term intake, may incur energetic and ecological costs in the medium and long term (Costa et al., 2024; Lima et al., 2018). For a restricted species whose persistence already depends on specific environmental conditions, the loss of these key processes may represent an additional threat to population viability (Agostinho et al., 2004; Petsch et al., 2023).

Our results reinforce that flood pulse alteration by dams such as Belo Monte exerts pressures that go far beyond physical habitat modification. By compromising the dynamics of food resource availability, flow regulation may generate physiological consequences for fish populations, especially those with restricted distributions, such as *Archolaemus janeae*. The loss of trophic diversity, the reduced quality and predictability of resources, and the need for forced dietary adjustments reveal a scenario of ecological vulnerability with potential cumulative effects on survival and recruitment. These findings highlight the urgent need to integrate the maintenance of ecologically adequate flow regimes into hydropower management policies. Strategies such as the implementation of environmental flows and continuous monitoring of fish trophic responses should be prioritized to mitigate the negative impacts of flow regulation and preserve the ecological functionality of endemic and range‐restricted species in rivers undergoing intense hydrological modification.

## AUTHOR CONTRIBUTIONS

Conceptualization: A.F.V.N.M.C., A.A.J.J., R.R.G., A.S.F., T.M.S.F. and L.F.A.M. Data collection: A.F.V.N.M.C., A.A.J.J., R.R.G., E.G.P. and T.M.S.F. Data analysis: A.A.J.J. and E.G.P. Writing – original draft: A.F.V.N.M.C., A.A.J.J., E.G.P., A.J.F.J. and A.S.F. Writing – review and editing: D.T.H., L.F.A.M. and T.M.S.F. Funding acquisition and supervision: L.F.A.M.

## FUNDING INFORMATION

Coordenação de Aperfeiçoamento de Pessoal de Nível Superior.

## CONFLICT OF INTEREST STATEMENT

The authors declare there are no competing interests.

## Data Availability

The data that support the findings of this study are available on request from the corresponding author. The data are not publicly available due to privacy or ethical restrictions.
